# The Native Bees of Lolland (Denmark) Revisited after 100 Years: The Demise of the Specialists

**DOI:** 10.3390/insects13020153

**Published:** 2022-01-31

**Authors:** Claus Rasmussen, Markus Arne Kjær Sydenham, Hans Thomsen Schmidt, Henning Bang Madsen

**Affiliations:** 1 Department of Agroecology, Aarhus University, 8830 Tjele, Denmark; 2 Norwegian Institute for Nature Research (NINA), 0855 Oslo, Norway; markus.sydenham@nina.no; 3 Independent Researcher, 7500 Holstebro, Denmark; htschmidt@outlook.dk; 4 Department of Biology, University of Copenhagen, 2100 Copenhagen Ø, Denmark; hbmadsen@bio.ku.dk

**Keywords:** conservation, functional traits, specialization, bee decline, pollen preference, floral resources

## Abstract

**Simple Summary:**

Bees are important pollinators of both cultivated plants and nature. Widespread reports exist about bee decline, but very few studies have looked at the changes to bee communities over a century. Revisiting an earlier study by a resident schoolteacher in Lolland, Denmark, we have determined several changes to a local bee community. Bees with a narrow food plant range and long-tongued bumblebees are less likely to still occur in the study area. We also examined the pollen collected by the bees a century ago. The majority of the pollen was from plants still encountered in the study area. Thus, the decline is likely linked to land-use changes and reduction in the abundance of available food plants rather than the complete extirpation of critical food plants. Such information is important for mitigating pollinator decline any further.

**Abstract:**

There is a global concern over insect declines, including both species loss and population declines. In particular, declines of species, such as bees that anchor trophic interactions and shoulder many of the essential ecosystem services, have been the focus of broad public concern. However, our understanding of what characterizes those species that are lost because of declines over long periods is limited by a scarcity of comparative studies. We here compare the species composition from a collection of bees sampled over two decades (2000–2019) from the island of Lolland in Denmark, with a collection also sampled over two decades (1900–1919), but a century ago by Jørgensen and his contemporaries. We further test if (1) the probability that bee species that were sampled a century ago are also found today depends on their degree of floral specialization; (2) and use information from pollen samples from bees from the historical records to assess if certain floral resources have been lost. In total, 203 species were recorded in the two periods, but less than half, 92 species, occurred in both sampling periods. A total of 174 species of bees were recorded from 1900–1919, and 121 species were recorded from 2000–2019, including 29 species not reported in 1900–1919. Notably, we report a reduction in the species composition among forage specialist bees from 26.4% to 15.7% of the bee fauna, a consistent and highly significant decline both when correcting for parasitic and social species. Pollen swabs from bees collected in the first period, 1900–1919, did not identify any plants that are not available today but documented a series of plants that were important to bees back then. These plants are still common today, such as *Taraxacum* and *Salix*. Our findings highlight the importance of local and historical faunistic studies, such as that of Jørgensen, who was a resident schoolteacher on the island of Lolland in southern Denmark, for documenting how changes over time affect the species composition in bee communities.

## 1. Introduction

The association between bees and flowers is one of the most famous examples of insect-plant interactions, and it is unquestionable that such interactions are of critical importance for terrestrial biodiversity. Bees can nest in a variety of substrates, including burrows in the soil and wood or plant piths [1]. However, whether the bees build their own nest or they are kleptoparasites that usurp the nests of other bees, their offspring almost invariably develop upon pollen and nectar. Some bees, in particular eusocial species that maintain their colony year round, are generalist pollen foragers, or polylectic, with no apparently specialized plant associations. Other groups of bees have more specific plant preferences, limited in range from one plant species only (strict monolectic) to plant species belonging to a couple or more genera in the same family (oligolectic) or across a narrow range of plant families (mesolectic) [1,2]. The close and often specific relationship with groups of plants makes bees one of the most important pollinator groups: a keystone taxon.

There is mounting concern over insect declines, including both species loss and population declines [3,4,5,6,7,8]. In particular, declines of keystone species, such as pollinators, are troubling given that they anchor trophic interactions and shoulder many of the essential ecosystem services of their respective communities [9]. Widely recognized threats to insect biodiversity, including pollinator diversity, are habitat destruction, agricultural intensification (including pesticide use), climate change, and invasive species, but also atmospheric nitrification from the burning of fossil fuels and the effects of droughts and changing precipitation patterns [9]. Indeed extinction rates of bees and wasps tend to increase with agricultural intensification [10].

The data evidence for pollinator decline is diverse, from global to local in scale, but most often based on past checklists and collections of biological specimens stored in natural history museums [11,12,13]. One of the most extensive local historical datasets is that of bee species collected by Charles Robertson near Carlinville, Illinois, USA (e.g., [14]). The study site was revisited after 75 years by John Marlin and Wallace LaBerge [15], who resampled only 65.4% of the Robertson species. They explained the difference in species numbers by the wider range of flower species targeted in the past by Robertson and noted an overall persistence of the bee fauna, despite a negative change in land-cover in the intervening period. However, less overlap was achieved when specifically the plant-insect interaction resampling was attempted, with an apparent degradation of interaction network structure and function [16]. Other studies have reconstructed and compared past faunas using a global data approach by mining historical museum records, either for widespread genera [17], the entire fauna of bees [8], or the diversity of pollen loads from museum specimens over time [12]. Observational data of bees [18,19] has also been used, with the general caveats that species-level observational records rarely span an entire century and historical museum records were often collected opportunistically for taxonomic diversity and display, and not for generating baseline data for later assessing changes in the bee fauna.

Conclusions from long-term surveys have mostly supported the findings by Biesmeijer et al. [18] that there is evidence of pollinator decline, most frequent among habitat and diet specialists, in univoltine species, and/or in non-migrants. The closest to a comprehensive assessment of long-term population trends in Denmark is the commented version of the national red-list [20]. Of the assessed species, 44% are of conservation concern (107 species) in one of the six categories: Regionally Extinct (RE), Critically Endangered (CR), Endangered (EN), Vulnerable (VU), Near Threatened (NT), and Data Deficient (DD). The endangered species, i.e., the Critically Endangered, Endangered, and Vulnerable, make up 23% of all of the red list assessed species in Denmark; that is, roughly a quarter of the species are at risk of facing negative population trends.

**Figure 1:**
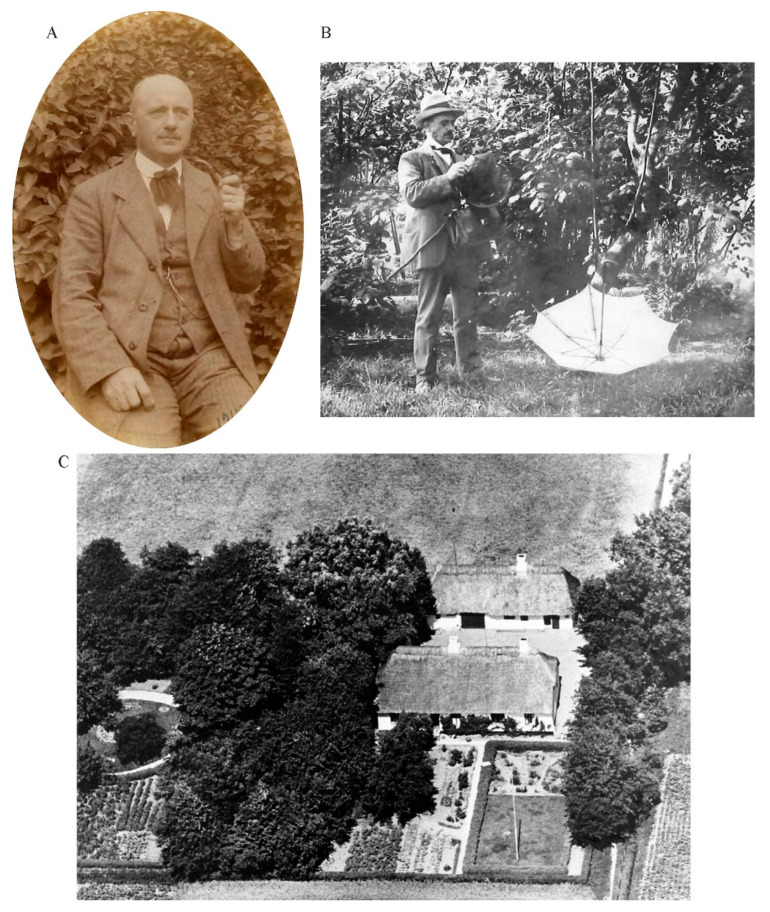
(**A**) Lavrids Jørgensen (1865–1937), photographed in 1915 by Peter Esben-Petersen. (**B**) Lavrids Jørgensen in the field surveying for insects, with a sweep net and umbrella (both images are kindly reproduced with permission from the archive of Entomology, Natural History Museum of Denmark, University of Copenhagen). (**C**) Strandby Skole (Strandby School) (54.668° N, 11.586° E) in an early image with a thatched roof. Notice the diversity of what are presumably vegetables and the diversity of microhabitats in the garden (image kindly reproduced with permission by Anders Thaarup and Christa Varming).

A particular important local faunistic study or baseline study of bees in Denmark was carried out by Jørgensen [21]. Hans Laurits Nicolai (“Lavrids”) Jørgensen (Figure 1A,B) was born on 29 December 1865 in Birket, on the northern parts of Lolland in Denmark. He graduated as a teacher from Skårup Seminarium, near Svendborg on Funen in 1885, and became the school principal at Strandby Skole (Strandby School) near Sandager and east of Nysted, Lolland in 1897 (Figure 1C). Following initial work on Lepidoptera (e.g., [22]), Diptera (e.g., [23]), Neuroptera and related taxa [24], Henriksen [25] note that Jørgensen in 1915 took on the little treated group of Danish bees for monographic treatment. The thought of studying the Danish bee fauna probably arose earlier when Jørgensen [26] wrote, “*Perhaps it could trigger some of the members of the Natural History Associations to engage in the study of the bees. Unfortunately, in Danish, we do not even have the hint of a work that can guide beginners in bee systematics*” (our translation). Jørgensen took on the task himself, resulting in a collection of bees, now historically important, from the vicinity of Strandby, a number of articles on bees [26,27,28,29,30,31,32,33,34], as well as the only Danish monographic study dedicated to the bees of Denmark [21]. The namesake and contemporary Danish entomologist, Peter (“Pedro”) Jørgensen (1870–1937) also known from the bee literature (e.g., [35,36,37]) only share a patronymic last name, and is not otherwise related to Lavrids [38].

We here re-examine the bee fauna of Lolland 100 years after Jørgensen. We assess the diversity now and then, compare the conservation status of the bees, the functional traits defining the different groups of bees, and the diversity of pollen sampled from bees collected by Jørgensen. Specifically, we ask if: (1) bees that were considered rare 100 years ago have been lost from the landscape; (2) if, as found by Biesmeijer and colleagues [18], the degree of floral specialization of bees dictate the probability that species, reported a century ago, occur in today’s bee fauna; (3) and if the flowers visited by bees 100 years ago are considered rare in today’s landscapes.

## 2. Material and Methods

### 2.1. Study Area

**Figure 2:**
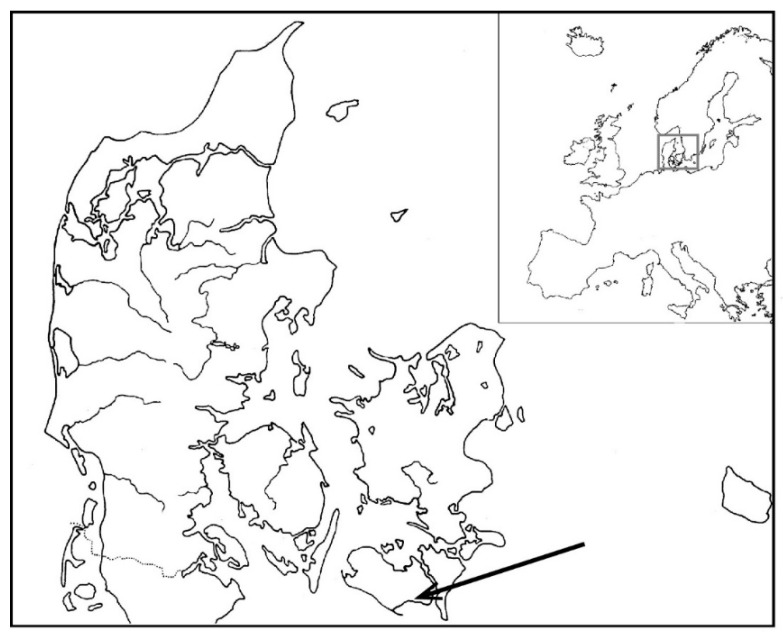
Denmark in the northern part of Europe. The location of Strandby on the island of Lolland is indicated with an arrow.

Lolland is the fourth largest island (1242 km^2^) in Denmark, surrounded by the Langelandsbelt, the Fehmarnbelt, the Baltic Sea, Guldborg Sund, and the Småland waters (Figure 2). The Guldborgsund municipality, where the school, Strandby Skole, near Sandager is located (54.668° N, 11.586° E), has an average temperature of 18.1 °C during the warmest month of the year in July (average 2006–2015), with a climate buffered by the close proximity to the sea preventing extreme temperatures year-round. The yearly average temperature in Guldborgsund is higher (9.3 °C) than the country average (8.9 °C), and rainfall is reduced because of the flat terrain that limits cloud formation [39]; thus, the physical settings favor the presence and persistence of warm-dependent insect species such as bees. At the same time, the entire south-facing coastline of Lolland is only narrowly separated from Germany by the Baltic Sea, making the island a known colonization point for southern climate migrants expanding northward [40]. Historical climate data from Denmark shows a higher corrected annual average temperatures for the period 2000–2019 compared to 1900–1919 [41]. In the recent 2000–2019 period, 19 out of 20 years are above the average temperature relative to a 1981–2010 baseline, while the early period from 1900–1919 had 18 out of 20 years below [41]. Annual accumulated precipitation was lower in 18 of 20 years in the 1900–1919 period compared to higher in 13 of 20 years in the 2000–2019 period [41]. In 1872, a disastrous flood struck large areas of Lolland, and in the following years, the areas west of Strandby were secured by effective dikes protecting inland areas but also extending the coastline and draining wetland areas [42]. However, the local area that Jørgensen later sampled probably remained largely unaltered at the time.

### 2.2. Collections

We first searched for all physical Lolland specimens collected during 1900–1919 and 2000–2019 in the collection of the Natural History Museum of Denmark, Copenhagen (ZMUC) and Natural History Museum in Aarhus, Denmark (NHMA). The historical period from 1900 to 1919 is defined chiefly by collections made by Jørgensen, with few additional specimens presumably collected and labeled by Anders C. Jensen-Haarup, Albert Kløcker, Jens P.O. Kryger-Jensen, Møller, P. Nielsen, and N. Petersen. Although it is also possible that some of those few specimens were caught by Jørgensen for exchange with entomologists who labeled the specimens using their name to indicate ownership. It is not possible to know from either publications or museum records the effort Jørgensen devoted to collecting and observing. Apparently, Jørgensen only preserved small series of an identified material or only collected small series while out in the field. Most of the Jørgensen specimens were collected during 1915–1916, with less activity during the rest of his active period from 1906–1919. Following the completion of his monographic publication, Jørgensen apparently gave up collecting and documenting the bee fauna of Lolland. There are very few individuals collected in 1920 or later by Jørgensen, including *Melitta leporina* from Jørgensen’s home in Strandby 16 (July 1923) where it had apparently not previously been collected and preserved. Bees were not collected during every single year of these periods, but every available specimen from the two periods was included in the study. The majority of the data collected on bees from 1900–1919 is from those specimens Jørgensen deposited in 1936 at ZMUC [25]. A few specimens collected by Jørgensen are deposited at the NHMA. Specimen data are also partly verified from the monograph Jørgensen published on bees [21], which includes species name, description, distribution, conservation status, and food plants, as well as further hand-written notes regarding the distribution. This data is now conserved at ZMUC [43]. Data on bees from 2000–2019 include intensive bee surveys during 5–9 July 2017 and 11–12 May 2019, in addition to casual collections throughout the entire period. Recent surveys did not strictly resample areas Jørgensen had frequented, as some of those had very little relevant nature left for sampling. Sites were primarily selected based on an initial visual inspection, knowledge of bee habitats, and proximity to some of those areas Jørgensen had visited. Collections included active searching with sweep net near flowers and over potential nest sites, but also using blue and yellow vane traps and yellow pan traps in 2000–2019. Identifications are based on the same reference material and references as we have used in the past and listed elsewhere [44,45,46,47,48,49,50,51,52]. Recently collected specimens are deposited in the collections of ZMUC, NHMA, and provisional private reference collections of the authors and collectors listed in the acknowledgments. All collection records include species name, the month of collection, and UTM-10 square; the latter a labeled 10 × 10 km square dividing the country [50,53,54].

### 2.3. Feeding Specialization and Historic Pollen Samples

We obtained information on functional traits, namely diet specialization and flight period, of all species through a literature survey [50,55,56,57,58,59,60], supplemented by measurements of total body length (BL) for female specimens from [61] and original measures. Flight period was based on regional records (including from [50]) and not general European records as some extend beyond what was recorded at a higher latitude in Denmark. In order to describe the floristic diversity and sampling routines at the time of Jørgensen, we examined pollen-loads from bees collected across Lolland in 1900–1919 (two specimens from respectively 1922 and 1923 were also included). Pollen samples were collected directly from one to three female specimens of each pollen-gathering bee species. Only specimens that were both collected from southern Lolland by Jørgensen or contemporaries and had visible pollen grains when examined under magnification were included. Once pollen loads were located, a small piece of Kaiser’s glycerol gelatin was applied to adhere to pollen grains with clean forceps and placed on a microscope slide. After melting (<50 °C) the gelatin, a coverslip was placed and slightly pressed to spread pollen grains on the slide before sealing the slip with a drop of nail polish. This largely follows the techniques outlined by Kearns and Inouye [62], except phenol and fuchsin for preservation or staining were omitted. Pollen samples were submitted to Quality Services International (QSI), Bremen, Germany, for identification to the lowest possible taxonomic level. For each sample corresponding to a single female individual, up to 100 pollen grains were counted and identified. This provided a relative presence of different pollen sources in each sample. Additional pollen types observed in the sample were recorded, but not counted. Insignificant pollen amounts contributing to <5% of the counts were excluded from our analysis as they may originate from contamination during handling at the time of capture or later. Due to age and unknown preservation techniques at the time of the collection of the bees, some of the pollen grains were too degraded to be properly identified. Other samples could not be properly identified due to convergent pollen morphology within the family or plant group. All pollen voucher specimens and pollen slides were labeled and deposited at ZMUC.

We used summary statistics from data tables processed in Excel and JMP114.0.0 to assess if species considered rare and endangered in 1900–1919 have been lost in today’s landscapes. We used logistic GLMs to test if the floral specialization of bees recorded in 1900–1919 affected the probability of them being found in today’s landscape (2000–2019). Because solitary bees show stronger responses than bumble bees to habitat loss at local scales [63], we tested the influence of floral specialization on the probability of bees being extant by building models for all bees, as well as exclusively for solitary bees and bumblebees. We tested if pollen generalists (polylectic bees) were more likely than pollen specialists (oligolectic bees) to be extant. Lecty status (presence/absence) does not capture the actual floral preferences of bees. We therefore also used detrended correspondence analysis (DCA) to position the 69 bees sampled in 1900–1919, and for which we had pollen samples in a four-dimensional space based on their similarities in scopal pollen content. The DCA was run in a presence/absence matrix with plant families as rows, and bee species as columns, depending on if pollen from a specific plant family had been recorded on the bee species. DCA scores of bee species reflected the floral preferences of bees so that species with similar scores were more ecologically similar than species with different scores. We extracted the species-specific DCA scores on the four DCA axes and tested if the probability of a bee being extant depended on the DCA scores on each axis. For bumblebees (*Bombus*), all species were classified as polylectic. A common ecological separation within bumblebees is between short and long-tongued species [64,65]. We, therefore, tested if bumblebee tongue length was related to the probability of bumblebees being extant. We also tested if the probability of bumblebees being extant depended on their DCA scores. For all models, we fit individual models for each variable (lecty status, DCA scores, and bumblebee tongue lengths) and tested the significance of each variable individually. We then included significant variables in a full model to test their marginal effects. We ran models testing DCA scores separately because we only had pollen samples, and therefore DCA scores, for a subset of the bee species. For statistical analyses of lecty, we excluded kleptoparasitic species because their presence reflects the availability of host-nests and not specific flowers in the same way as for their hosts. We used DHARMa residual plots to test that residuals met model assumptions. All of the latter analyses were run using RStudio [66] and R packages [67,68,69,70,71].

### 2.4. Status

The International Union for Conservation of Nature (IUCN) categories of threat are widely used in ‘Red lists’ of endangered species and are an important conservation tool at international, national, and regional levels. The entire Danish bee fauna was recently red-listed, providing a starting point for measuring changes in the fauna [20]. Prior assessments for the Danish fauna are only available for bumblebees (*Bombus* spp.) [72]. Jørgensen provided a tentative status for all bee species in his monograph [21]. Categories used by Jørgensen are listed in Table 1. These are pre-IUCN and while not *per se* assessing the risk of extinction, they do provide a measure of how likely the species were to be encountered at the time or if already considered regionally extinct. We use these two different assessments, made during the end of the first period from 1900–1919 and during the end of the second period from 2000–2019, as a proxy for the species abundance. The category ‘Not applicable’ (NA) was used for 48 species in the recent IUCN-based red list of Danish bees [20], and while that includes species under establishment (i.e., a species had been resident in Denmark for less than 10 years), as well as species found only in the form of stray individuals (not resident in Denmark), these would all likely fall under the category ‘very rare’ in the terminology of Jørgensen.

## 3. Results

### 3.1. Collections

**Figure 3:**
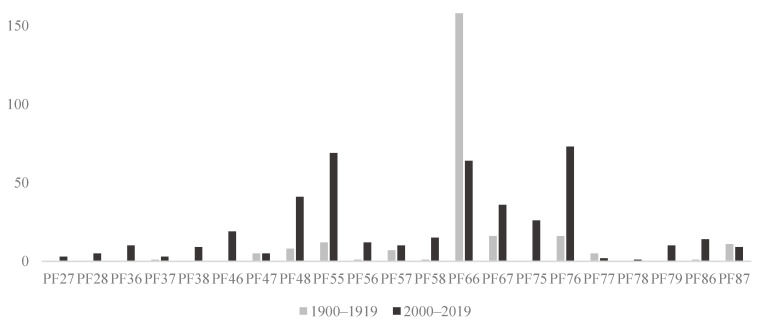
Number of species of bees from Lolland recorded from different UTM-10 localities in each of two periods; 1900–1919 and 2000–2019.

**Figure 4:**
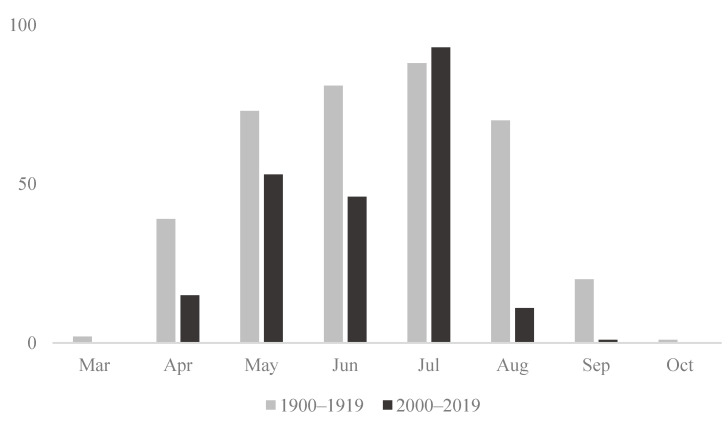
Number of species of bees from Lolland recorded during different months of the year in two periods; 1900–1919 and 2000–2019.

In total, 203 species (Table 2 and Supplemental Table S1) were reported in the two periods covering 1900–1919 and 2000–2019, but less than half, 92 species, occurred in both sampling periods. Jørgensen and his contemporaries recorded 174 species of bees from Lolland in the period from 1900–1919 (see Supplemental Table S1 legend for notes about certain of the species). Our recent efforts discovered 121 species of bees from Lolland, including 29 species not reported in 1900–1919 (see also [52]). At the same time, 82 species reported in 1900–1919 were not re-discovered in 2000–2019 (Table 2). The majority of the 1128 specimens from Lolland 1900–1919 were collected by Jørgensen himself. Only *Andrena nigroaenea* and *Anthophora plumipes* are represented by >30 specimens collected by Jørgensen, while 71 species are represented by merely one or two individuals. In the recent period (2000–2019), we recorded 1409 specimens of bees, with the majority collected during 2017 and 2019. It is evident that Jørgensen and contemporaries mostly collected around the UTM-10 square PF66 (where his home in Strandby is located), whereas in recent collections, both PF66 as well as bordering squares, have been visited (Figure 3). Being a resident entomologist on Lolland, Jørgensen had the advantage—besides strong local knowledge of collecting localities—of extending the active collecting period to the extremes of the season (Figure 4). In particular, Jørgensen was able to sample during the spring and the fall, whereas the recent collections from visiting entomologists were timed to coincide with peak adult bee activity periods, May to July, missing out the early spring (April) and fall (August–September) (Figure 4).

### 3.2. Feeding Specialization and Historic Pollen Samples

Strong differences were found when comparing ecological and functional traits for bees collected during the two periods (Table 3). In the recent survey, many of the newly documented species for Lolland (species unique to 2000–2019) were kleptoparasitic (Table 3). Many of the species that have not been recorded from Lolland since the first period were the forage specialists (oligolectic). That is, they exhibit a narrow preference for pollen sources, as opposed to the polylectic species that utilize pollen from multiple different plant families [2]. Across both periods and all species collected, 24.6% were oligolectic, but of the species unique to the 1900–1919 period, 37.8% were oligolectic, as opposed to 13.8% among the species unique to the 2000–2019 period. Voltinism was less marked, but more of the species unique to 1900–1919 was univoltine and thus specialized in time with one generation per year, as opposed to other groups (Table 3). While social bee species also establish nests from a single founder, the provision and colony growth are very different from a solitary nest-building species [73]. We, therefore, report results both for all species and all species excluding the non-resident species (i.e., NA category species from IUCN, often stray individuals), parasitic species, and the social bumblebees (*Bombus* spp.) and honeybees (*Apis mellifera*) (Table 3). A total of 41.4% of the bee species unique to 2000–2019 were parasitic, more than twice as many as 1900–1919. If kleptoparasitic species, social bumblebees (*Bombus* spp.) and honeybees (*Apis mellifera*) altogether were removed, all species unique to 2000–2019 were univoltine, but the general pattern of feeding specialization remained across the different groups.

**Figure 5:**
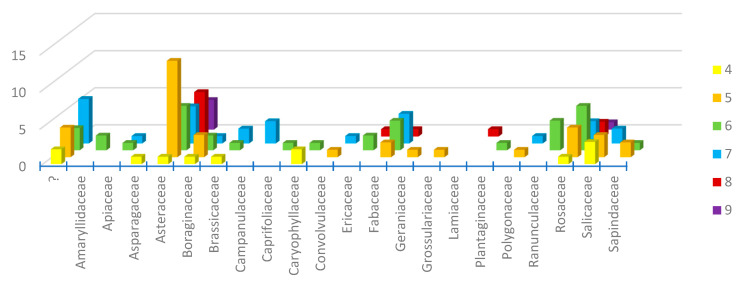
Pollen from the following plant families was recorded from individual bees collected during April (4) to September (9) in the early period from 1900–1919.

We were able to recover and identify pollen from 86 of the 92 female individuals collected in 1900–1919 by Jørgensen or contemporaries. These individuals represent 80 different bee species. Pollen came from 21 different plant families represented by 65 different pollen types (one type can represent multiple species, but multiple types cannot represent one species), with a particular abundance of both the number of pollen grains and occurrences in the samples from Asteraceae, Rosaceae, Fabaceae, Salicaceae, and Boraginaceae. The most commonly observed genera were *Taraxacum* and *Salix* (14 and 8 individuals, respectively). Most diversity of pollen was found on *Andrena bicolor* (eight different pollen types) and *Lasioglossum leucozonium* (five different pollen types), both known polylectic species (Supplemental Table S1). Looking at the resources throughout the season, it is evident that Jørgensen followed the seasonal flowers when sampling bees (Figure 5)—briefly described as spring with the many willow specialists (*Salix* in Salicaceae) and few other resources until mid-April. Early summer with many composites (Asteraceae), followed by mid-summer groups like legumes (Fabaceae), and then finally late summer flowers, like Ericaceae.

**Figure 6:**
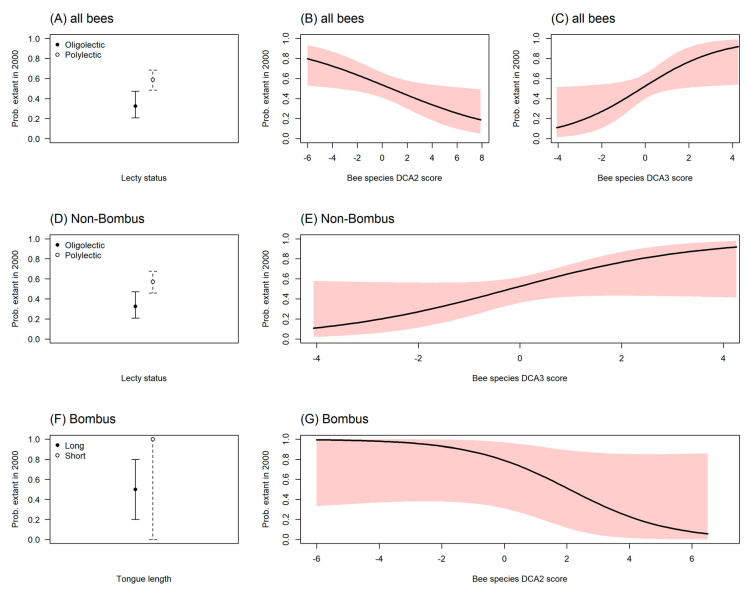
Probability for non-parasitic species recorded in the 1900–1919 period to be extant in the 2000–2019 period. (**A**) Based on lecty (feeding), oligolectic species were less likely to be extant (*p* < 0.01). (**B**) Probability for bee species being extant decreased with DCA2 floral preference scores (Supplemental Figure S1 and Table S2) (*p* < 0.05). (**C**) Probability for bee species being extant increased with DCA3 floral preference scores (Supplemental Figure S1 and Table S2) (*p* < 0.05). (**D**) Based on lecty, oligolectic species were less likely to be extant when *Bombus* species were excluded (*p* < 0.01). (**E**) For Non-*Bombus* species, floral preferences (DCA3 scores) influenced the probability of being extant (Supplemental Figure S1 and Table S2). *Bombus* species were excluded (*p* < 0.05). (**F**) Based on tongue length [74]. Higher probability for short-tongued *Bombus* species than for long-tongued *Bombus* species (*p* < 0.05). (**G**) Probability for *Bombus* species being extant decreased significantly, with species DCA2 scores (Supplemental Figure S1 and Table S2) (*p* < 0.05), but the effect size was not statistically significant (z-value = −1.6, *p* = 0.11). Parasitic solitary and Cuckoo bumblebees (*Bombus (Psithyrus)*) were excluded from all analyses excluded. Tables including *p*-values are provided in Supplemental Table S2.

Using binomial GLMs, our analysis showed a significant difference (χ^2^ = 8.54, df = 1, *p* < 0.01 for all bees, see Supplemental Tables S2 and S3) in the persistence of polylectic species, as opposed to those that are oligolectic (Figure 6A), this is both for all non-parasitic bees and for all non-parasitic and non-*Bombus* species (Figure 6A,D). In addition, we found that the probability for bee species being extant increased (χ^2^ = 4.84, df = 1, *p* < 0.05, see Supplemental Tables S2 and S3) when they exhibited higher DCA3 floral preference scores (e.g., Apicaeae, Polygonaceae, Grossulariaceae, Caprifoliaceae, Rosaceae in Supplemental Figure S1) for both non-parasitic and non-*Bombus* species (Figure 6B,C,E). Similar, there was a lower probability (χ^2^ = 4.96, df = 1, *p* < 0.05) of being extant for the long-tongued *Bombus* species [74] (Figure 6F,G).

### 3.3. Status

The distribution of red list categories for the bee species of Lolland is listed in Table 4. A summary is also provided for those species unique to each of the two survey periods. Status is according to both Jørgensen [21] and Madsen [20]. As Jørgensen did not assess all taxa now known from Lolland, some species are missing, and for comparison, numbers from the table are reported as a percentage of the total number of species assessed. Of the species only found during the early period, Jørgensen considered 79% of these less than common (Table 1 and Supplemental Table S1), opposed to 50% less than common on the current Danish red list (RE + CR + EN + VU + NT). However, of the species only found in 1900–1919, as many as 26% of the species were now considered NA in the Danish red list, mostly because they are stray individuals, under establishment, and not residents. Jørgensen did not have a NA category but presumably considered these CR and EN (18 of the 20 species he assessed CR + EN are now NA), realizing it was uncommon to encounter these species. In the recent survey, there were two NA species, both considered under establishment within the last ten years (*Lasioglossum pauxillum* and *Epeoloides coecutiens* [75,76]). Of the 29 species found only in the recent survey, 10% are red-listed and none in the higher categories of CR + EN. Of the LC Danish species, five are red-listed on the European red list (*Colletes fodiens* (VU), *Andrena hattorfiana* (NT), *Lasioglossum quadrinotatum* (NT), *Bombus muscorum* (VU), and *Epeolus cruciger* (NT)) [77].

## 4. Discussion

We have revisited and sampled an area with a rich historical record of bees. After 100 years, we can report a reduction in the species richness of forage specialist bees from 26.4% to 15.7% of the fauna, a consistent decline both when correcting for parasitic species, social species, and non-resident species. However, based on the similarity indices for the two periods, there is a rather high turnover considering that sampling is from the same general area. The decline of forage specialists is apparent when the entire bee fauna is analyzed, but also when correcting for life history by excluding parasitic bees, bumblebees, and honeybees. Specialist bees are only found in habitats with ample amounts of their forage plants [1]; if these plants vanish or become too sporadic to provide a reliable food resource, the bees will also disappear. We are unaware of botanical surveys addressing such changes near Strandby and the remaining study area. Even more at risk are kleptoparasitic bees specialized in Denmark on one or more specialist bees, such as *Nomada armata* (NT) on *Andrena hattorfiana* and the now regionally extinct *Nomada argenata* (RE) on *A. marginata*. In turn, polylectic species can persist on a wide range of food plants, and in the process of land-use changes, these bee species are less likely to disappear provided sufficient alternative resources. If polylectic species require a broad but very specific range of hosts, they may also become vulnerable. Our main concern is the apparent reduction among the forage specialists. While the functional diversity of pollination networks may be buffered by polylectic species [78], including maintenance of diversity in plant communities, the loss of specialists is an indicator of an impoverished nature and a homogenization of the landscape. Loss of specialist pollinators is apparently global [16], across multiple insect groups [6], where the specialists have been identified as particularly vulnerable [79].

Pollen records are consistent with current conditions and do not point to an explanation alone. *Salix* and *Taraxacum* were the most prevalent pollen types in the analysis of historical specimens and were also the two most prevalent pollen types reported in a recent study [50]. Plant genera frequented by those specialist bees that were not resampled from Lolland in 2000–2019 include *Achillea*, *Allium*, *Brassica*, *Campanula*, *Echium*, *Lotus*, *Salix*, *Sinapis*, *Taraxacum*, *Trifolium*, and *Vicia*. All plant taxa that are still present and available in the local area [80] and at least partially revisited during the 2000–2019 surveys. While present, they may have changed in abundance or somehow no longer in numbers supporting a sustainable population of some of those species. That is unclear.

Once again, a decline in long-tongued bumble bees is detected [81]. It has been suggested that these often late-emerging species, specialized in gathering pollen from Fabaceae, have all declined due to the loss of unimproved species-rich grasslands, among others [82]. In particular, species that have disappeared, such as *B. distinguendus*, *B. ruderatus*, and *B. veteranus* must have thrived on extensive fields of red clover for foraging [83,84].

Did parasitic bee species increase in species richness, as our data suggest? This could be an artifact due to our diversified collecting strategies, as we, in addition to sweep netting over flowers and nests, and passive trapping methods that may have sampled a different community of bees. Passive traps were not applied during the time of Jørgensen, although he would have spent more man-hours in the field and thus should have encountered and collected what was even rare at the time. Jørgensen, in the early twentieth century, faced a lack of public transportation and a scarcity of private automobiles. Sites were probably visited on foot. If a collecting artifact, it would likely reflect also in other groups of bees. It could also be an artifact during identification. Due to taxonomic similarity among species, Jørgensen may have overlooked some of the diversity while retaining only a short series of individuals. Lastly, it should be tested if kleptoparasitic bees are spreading faster northward than other bees, tracing already established hosts, while following a warmer climate [85]? To consider potential bias, all assessments were done both with and without parasitic bees.

Differences in the sampling method are outlined in the material and methods section and may have affected the results. The lack of sampling from the extremes of the season may have missed species during the recent surveys. However, the phenology of none of the species from Table 2 finishes before April–May [50], and of the potentially missing autumnal species, only *Hylaeus cornutus*, *Hylaeus difformis*, and *Melitta nigricans* have a late flight period in Denmark with records exclusively from August or later [50]. All three are NA species and should not be expected regularly in Denmark [20]. Both end-periods are scarcely surveyed recently. Another concern is whether we documented the bee fauna in 1900–1919 properly? Could it be that the differences we here report are even more marked? It is possible that Jørgensen could have misidentified some of the species he examined, and while only retaining short series of each putative species, he may potentially have discarded specimens representing additional species not recognized at the time. Several species in his collection have only been defined as good biological species after the period of Jørgensen, e.g., *Andrena gelriae* [86] or were erroneously considered conspecific by Jørgensen, e.g., *Nomada baccata* Smith [87] and *N. alboguttata* Herrich-Schäffer [88].

Jørgensen also encountered many species in ‘forests’ (based on locality names ending in “–skov”), with the vast majority of species from Keldskov in close proximity to Strandby school. Some 66 species are recorded from a locality with ‘forest’ in the name, 12 exclusively in ‘forests’. Of these 12 species, 7 were not reported in the 2000–2019 surveys. Today Keldskov, in particular, is a closed canopy forest, and no bees were seen inside the forest during our visits. Either Jørgensen only sampled from the edge, or the forest should have looked very different and open to light 100 years ago?

Is it still possible to sample as many bee species (158) from a small 10 × 10 km (UTM-10 PF66) square in Denmark as Jørgensen did? In recent surveys (2017, 2019), we did not see sufficient diversity of the current landscape to expect such a number locally and believe high numbers are only possible if sampling from a larger area. Although land management may have driven a steady decline, we also do notice interesting species unique to the 2000–2019 period. Several specimens of the Critically Endangered and oligolectic *Osmia niveata* were recorded from the very courtyard of Strandby Skole (2017). Jørgensen never reported it from Lolland during 1900–1919 and listed it, at the time, as Endangered (Table 1 and Table 2). In comparison, Robertson in Carlinville, Illinois, collected across the county in a one-horse buggy over unimproved roads [15], but Jørgensen appears to have been further limited to localities within a few kilometers of walking or biking distance from his school. Primarily based on an assessment of habitat quality that deteriorated and fragmented across most of Lolland, recent surveys ventured further away from the Strandby area. By expanding away from the immediate surroundings of Strandby, we may have recorded additional species than those only in the Strandby vicinity.

While other studies tracing changes in the bee fauna (e.g., [8,17]) have focused on rigorous analysis of specimen level data, we do not feel this would be appropriate for the present dataset. Historical specimen level data is often severely biased because collectors are already knowledgeable about the taxonomy of their objects, and sampling is “cherry picking”, by selectively excluding long series of the most common taxa from samples while at the same time targeting rare and conspicuous species (Doug Yanega, pers. comm.; [89]). In the recent period from 2000–2019 a number of collections were made with passive bulk sampling protocols, such as pan trapping. This resulted in large sample sizes of the most common species, but for logistical reasons, only representative specimens of the common species have been prepared and recorded fully. Because of this focus on diversity, while both collecting and curating samples, data necessarily show an overrepresentation of the less common species. These biases in collection-based data are difficult to account for, although modeling techniques are becoming more sophisticated [90]. Instead of specimen-level data, presence-based red-list data can be used, as we did, in order to assess the frequency of the different bee species.

In conclusion, there has been a loss of forage specialists among bees in this study. Many of the plants they are specialized in are still available, but nevertheless, some of the species have disappeared or become more restricted [80]. Many more non-resident species (NA category) were reported by Jørgensen. Either because they were more likely to be seen by the resident entomologist or because they were more abundant earlier in the source areas and thus more likely to be transported to Lolland and Denmark. Obvious explanations for the loss of forage specialists are the transition from smaller to larger agricultural fields, with loss of hedges, drainage, overgrazing, and even changing practices such as abandoning haymaking. Even the abandoning of former earthen walls and thatched roofing has reduced the number of possible nesting places for many bee species, including *Hylaeus pictipes*, not found now but considered not rare (NT) at the time of Jørgensen. While some of the bees have disappeared, others have newly migrated to Lolland. This includes mostly polylectic species. While Lolland may host some special conditions for early arrivals to Denmark of species, Lolland is similar to the rest of the country densely cultivated and traversed by roads and cities. What Lolland shows us is likely applicable for the remaining parts of the country, but we have little extensive baseline data, such as the data from the vicinity of Strandby School. Likely it is not a suite of plants that has since completely disappeared, as our pollen data suggests; it is rather the many microhabitats providing nesting space and food that has vanished from the landscape.

## Figures and Tables

**Table 1:** Categories of status by Jørgensen [21] in Danish, translated to English and interpreted according to the International Union for Conservation of Nature (IUCN) classification.

Danish Category [21]	English [21]	IUCN Interpretation	IUCN
“forsvundet”	“disappeared”	RE	Regionally Extinct
meget sjælden	very rare	CR	Critically Endangered
Sjælden	rare	EN	Endangered
temmelig sjælden	rather rare	EN	Endangered
noget sjælden	somewhat rare	VU	Vulnerable
ikke sjælden	not rare	NT	Near Threatened
ikke almindelig	not common	NT	Near Threatened
temmelig almindelig	rather common	LC	Least Concern
almindelig	common	LC	Least Concern
meget almindelig	very common	LC	Least Concern

**Table 2:** Number of species from Lolland; summary of Supplemental Table S1.

	Bee Species Collected
Total no. spp. in both studies	203
No. spp. 1900–1919	174
No. spp. 2000–2019	121
No. spp. unique to 1900–1919	82
No. spp. unique to 2000–2019	29
No. spp. common to both periods	92
Jaccard similarity index for two periods	0.453
Sørensen similarity index for two periods	0.624
Total no. spp. in both studies, excluding *Apis, Bombus* and parasite species	138
No. spp. unique to 1900–1919, excluding *Apis, Bombus* and parasitic species	64
No. spp. unique to 2000–2019, excluding *Apis, Bombus* and parasitic species	16

**Table 3:** Traits of the bees collected during the two periods from 1900–1919 and 2000–2019. Values are the percentage (%) of the total number of species in the different periods.

		Both Periods	1900–1919	2000–2019	Unique to 1900–1919	Unique to 2000–2019	Common for Both Periods
Parasitic		24.6	21.8	29.8	17.1	41.4	26.1
Feeding preference							
	Oligolectic	24.6	26.4	15.7	37.8	13.8	16.3
	Polylectic	50.7	51.7	54.5	45.1	44.8	57.6
Excluding ‘Not applicable’ (NA)							
	Oligolectic	21.2	22.4	16.1	31.1	14.8	16.5
	Polylectic	52.0	53.3	54.2	47.5	44.4	57.1
Excluding *Bombus*, *Apis* and parasitic bees:							
	Oligolectic	36.2	37.7	25.7	48.4	25.0	25.9
	Polylectic	63.8	62.3	74.3	51.6	75.0	74.1
Excluding ‘Not applicable’ (NA), *Bombus*, *Apis* and parasitic bees:							
	Oligolectic	29.0	29.6	22.9	39.6	25.0	22.4
	Polylectic	71.0	70.4	77.1	60.4	75.0	77.6
Voltinism							
	Univoltine	89.7	89.7	86.0	95.1	89.7	84.8
	Bi- or multivoltine	10.3	10.3	14.0	4.9	10.3	15.2
Excluding ‘Not applicable’ (NA), *Bombus*, *Apis* and parasitic bees:							
	Univoltine	93.9	93.0	94.0	93.8	100.0	92.5
	Bi- or multivoltine	6.1	7.0	6.0	6.3	0.0	7.5
Average body length (mm)		10.6	10.7	10.6	10.6	10.0	10.8
Excluding *Bombus*, *Apis*, and parasitic bees:		9.7	9.9	9.3	10.2	8.4	9.6

**Table 4:** Conservation status of all species recorded in 1900–1919 and 2000–2019, followed by assessment for the species only found in 1900–1919 and in 2000–2019, respectively. Abbreviations are for Regionally Extinct (RE), Critically Endangered (CR), Endangered (EN), Vulnerable (VU), Near Threatened (NT), and Data Deficient (DD). Assessments are from both Jørgensen [21] and Madsen [20] and follow the IUCN categories from Table 1. Red listed is the sum of all those categories (RE + CR + EN + VU + NT). Jørgensen did not use anything to describe NA, which was excluded from his assessments. RE is also excluded, as Jørgensen did not have a historical baseline from which extinction could be inferred. None of the species from the survey are DD and therefore missing.

	Both Periods		Only 1900–1919		Only 2000–2019	
	Jørgensen	Madsen	Jørgensen	Madsen	Jørgensen	Madsen
RE	-	10 (5%)	-	10 (12%)	-	(0%)
CR	29 (14%)	8 (4%)	20 (24%)	6 (7%)	4 (14%)	(0%)
EN	27 (13%)	10 (5%)	17 (21%)	9 (11%)	1 (3%)	(0%)
VU	13 (6%)	12 (6%)	6 (7%)	10 (12%)	(0%)	1 (3%)
NT	48 (24%)	14 (7%)	22 (27%)	6 (7%)	3 (10%)	2 (7%)
Redlisted	117 (58%)	54 (27%)	65 (79%)	41 (50%)	8 (28%)	3 (10%)
LC	57 (28%)	125 (62%)	10 (12%)	20 (24%)	5 (17%)	24 (83%)
NA	-	24 (12%)	-	21 (26%)	-	2 (7%)

## Data Availability

Data made available in supplemental section.

## Supplementary Materials

The following supporting information can be downloaded at: https://www.mdpi.com/article/10.3390/insects13020153/s1, Table S1: bee species recorded; Table S2: tables of *p*-values; Table S3: tables of summary output; Figure S1: pollen samples and their DCA scores.
