# Supplementary material for: The Native Bees of Lolland (Denmark) Revisited after 100 Years: The Demise of the Specialists
Source: Insects. 2022 Jan 31;13(2):153. doi: 10.3390/insects13020153 (PMC8880802; doi:10.3390/insects13020153)
Supplement: Supplementary file 1 [file insects-13-00153-s001.zip › insects-1541553-supplementary.pdf]

Table S1. Species of bees recorded from Lolland, Denmark, during the sampling periods 1900-1919 and 2000-2019, as well as conservation status according to both Jørgensen [21] and Madsen [20]. Last column is whether a direct feeding specialization (oligolectic/polylectic) or parasitic. Bumble bees (Bombus) have been annotated as long-tongued (LT) or short-tongued (ST). Older confirmed records from Lolland exist for *Andrena schencki* [81], but it had disappeared when Jørgensen [21] wrote his monograph. *Megachile lagopoda* may have been historically present on Lolland [21,28,43], but we have not located the source of the information and consider it a misidentification. There are no specimens in ZMUC from Lolland of this large species. Outside these two sampling periods the only species other than *A. schencki* reported from Lolland is *Osmia leaiana* [82]. Two of the species reported from Lolland by Jørgensen [21] were excluded due to absence of physical vouchers. *Colletes marginatus* [as *Colletes balteatus*] reported from Brunddragene on Lolland is likely a misidentification [83], as the only record of *Colletes* from this locality is a single individual of *C. daviesanus* [collected July 6, 1916]. A*ndrena ruficrus* [as *Anthrena rufitarsis*] from Strandby Skole may also have been a misidentification as there are no preserved specimens of this species from Lolland in the collection. Eleven of the species reported by Jørgensen as "*known from all parts of the country*" [our translation] were actually not seen from Lolland for the period 1900-1919. Despite the broad statement in the monograph, the following species are excluded for that period because of lack of physical vouchers: *Andrena helvola, A. tarsata, Dufourea dentiventris, Lasioglossum morio, L. parvulum, Bombus jonellus, B. subterraneus, B. sylvestris, Epeolus variegatus, Nomada fulvicornis* and *Nomada integra.* Nine species were present among the Jørgensen physical vouchers from Lolland 1900-1919 at ZMUC, but not listed from Lolland in Jørgensen [21], either because no specific locality from Lolland was provided in the monograph or because they had been misidentified: *Colletes succinctus*, *Andrena clarkella, A. ovatula, Panurgus calcaratus*, *Lasioglossum fratellum, L. punctatissimum, Bombus lucorum, B. ruderatus,* and *Nomada roberjeotiana.* These are included in this study. In addition, ten more species were identified from the physical vouchers, but were not recognized as valid species, or for other reasons not included in the monograph by Jørgesen [21]: *Andrena apicata, A. gelriae, A. intermedia, A. niveata, A. subopaca, Lasioglossum lativentre, L. semilucens, Sphecodes ferruginatus, S. geoffrellus,* and *Melitta tricincta.* These are also included in this study.

	1900-1919	2000-2019	Jørgensen	Madsen	Feeding
**Colletidae**					
Colletes cunicularius	x	x	(EN)	LC	Oligolectic
Colletes daviesanus	x	x	(NT)	LC	Oligolectic
Colletes fodiens	x	x	(NT)	LC	Oligolectic
Colletes marginatus		x	(CR)	NT	Oligolectic
Colletes similis	x	x	(CR)	LC	Oligolectic
Colletes succinctus	x		(LC)	LC	Oligolectic
Hylaeus brevicornis	x	x	(NT)	LC	Polylectic
Hylaeus communis	x	x	(NT)	LC	Polylectic
Hylaeus confusus	x	x	(LC)	LC	Polylectic
Hylaeus cornutus	x		(CR)	NA	Polylectic
Hylaeus difformis	x		(CR)	NA	Polylectic
Hylaeus hyalinatus	x	x	(NT)	LC	Polylectic
Hylaeus incongruus	x		(LC)	LC	Polylectic
Hylaeus pectoralis		x		LC	Polylectic
Hylaeus pictipes	x		(NT)	CR	Polylectic
Hylaeus punctulatissimus	x		(CR)	NA	Oligolectic
Hylaeus rinki	x		(CR)	RE	Polylectic
Hylaeus sinuatus	x		(CR)	RE	Polylectic
**Andrenidae**					
Andrena apicata	x			LC	Oligolectic
Andrena argentata	x		(VU)	EN	Polylectic
Andrena barbilabris	x		(NT)	LC	Polylectic

Andrena bicolor	x	x	(LC)	LC	Polylectic
Andrena carantonica	x	x	(LC)	LC	Polylectic
Andrena chrysopyga	x		(EN)	VU	Polylectic
Andrena chrysosceles	x	x	(CR)	LC	Polylectic
Andrena cineraria	x	x	(EN)	LC	Polylectic
Andrena clarkella	x	x	(NT)	LC	Oligolectic
Andrena coitana	x		(NT)	EN	Polylectic
Andrena curvungula	x		(CR)	NA	Oligolectic
Andrena denticulata	x		(NT)	LC	Oligolectic
Andrena flavipes	x	x	(NT)	LC	Polylectic
Andrena fucata	x	x	(NT)	LC	Polylectic
Andrena fulva		x		LC	Polylectic
Andrena fulvago	x		(LC)	VU	Oligolectic
Andrena fuscipes		x	(LC)	LC	Oligolectic
Andrena gelriae	x			RE	Oligolectic
Andrena gravida	x	x	(CR)	NT	Polylectic
Andrena haemorrhoa	x	x	(LC)	LC	Polylectic
Andrena helvola		x	(NT)	LC	Polylectic
Andrena humilis	x		(NT)	NT	Oligolectic
Andrena intermedia	x			NA	Oligolectic
Andrena labialis	x		(NT)	LC	Oligolectic
Andrena labiata	x	x	(VU)	LC	Polylectic
Andrena lapponica	x		(NT)	LC	Oligolectic
Andrena lathyri	x		(NT)	VU	Oligolectic
Andrena minutula	x	x	(LC)	LC	Polylectic
Andrena morawitzi	x		(EN)	EN	Polylectic
Andrena nigriceps	x	x	(VU)	LC	Polylectic
Andrena nigroaenea	x	x	(LC)	LC	Polylectic
Andrena nigrospina	x	x	(LC)	NT	Polylectic
Andrena nitida	x	x	(EN)	NT	Polylectic
Andrena niveata	x			RE	Oligolectic
Andrena praecox	x	x	(LC)	LC	Oligolectic
Andrena proxima	x		(CR)	NA	Oligolectic
Andrena semilaevis	x	x	(LC)	LC	Polylectic
Andrena subopaca	x	x		LC	Polylectic
Andrena thoracica	x		(EN)	EN	Polylectic
Andrena tibialis	x		(NT)	LC	Polylectic
Andrena varians	x		(LC)	NT	Polylectic
Andrena wilkella	x	x	(LC)	LC	Oligolectic
Panurgus banksianus	x		(LC)	LC	Oligolectic
Panurgus calcaratus	x		(LC)	LC	Oligolectic
**Halictidae**					
Dufourea minuta	x		(EN)	RE	Oligolectic
Halictus compressus	x		(NT)	RE	Polylectic
Halictus confusus		x		LC	Polylectic
Halictus maculatus	x		(CR)	CR	Polylectic
Halictus quadricinctus		x	(CR)	VU	Polylectic
Halictus rubicundus	x	x	(LC)	LC	Polylectic

Halictus sexcinctus	x		(CR)	NA	Polylectic
Halictus tumulorum	x	x	(LC)	LC	Polylectic
Lasioglossum albipes	x	x	(LC)	LC	Polylectic
Lasioglossum calceatum	x	x	(LC)	LC	Polylectic
Lasioglossum costulatum	x		(EN)	NA	Oligolectic
Lasioglossum fratellum	x		(CR)	LC	Polylectic
Lasioglossum laevigatum	x		(CR)	NA	Polylectic
Lasioglossum lativentre	x	x		NT	Polylectic
Lasioglossum leucopus	x	x	(LC)	LC	Polylectic
Lasioglossum leucozonium	x	x	(LC)	LC	Polylectic
Lasioglossum lucidulum		x		LC	Polylectic
Lasioglossum malachurum	x		(VU)	NA	Polylectic
Lasioglossum minutissimum	x	x	(NT)	LC	Polylectic
Lasioglossum morio		x	(LC)	LC	Polylectic
Lasioglossum nitidiusculum	x	x	(LC)	NT	Polylectic
Lasioglossum nitidulum	x		(EN)	VU	Polylectic
Lasioglossum parvulum		x	(NT)	LC	Polylectic
Lasioglossum pauxillum		x		NA	Polylectic
Lasioglossum punctatissimum	x	x	(CR)	LC	Polylectic
Lasioglossum quadrinotatum	x	x	(LC)	LC	Polylectic
Lasioglossum rufitarse		x		LC	Polylectic
Lasioglossum semilucens	x	x		LC	Polylectic
Lasioglossum sexnotatum	x		(NT)	RE	Polylectic
Lasioglossum sexstrigatum		x		LC	Polylectic
Lasioglossum villosulum	x	x	(NT)	LC	Polylectic
Lasioglossum xanthopus	x		(NT)	VU	Polylectic
Lasioglossum zonulum	x		(NT)	EN	Polylectic
Rophites quinquespinosus	x		(VU)	RE	Oligolectic
Sphecodes crassus	x	x	(NT)	LC	[Parasite]
Sphecodes ephippius	x	x	(EN)	LC	[Parasite]
Sphecodes ferruginatus	x			CR	[Parasite]
Sphecodes geoffrellus	x	x		LC	[Parasite]
Sphecodes gibbus	x	x	(LC)	LC	[Parasite]
Sphecodes miniatus		x		LC	[Parasite]
Sphecodes monilicornis	x	x	(NT)	LC	[Parasite]
Sphecodes pellucidus	x	x	(LC)	LC	[Parasite]
Sphecodes puncticeps	x	x	(NT)	LC	[Parasite]
Sphecodes reticulatus	x		(NT)	LC	[Parasite]
**Melittidae**					
Dasypoda hirtipes	x	x	(NT)	LC	Oligolectic
Macropis europaea	x	x	(VU)	LC	Oligolectic
Macropis fulvipes	x		(EN)	NA	Oligolectic
Melitta haemorrhoidalis	x		(LC)	LC	Oligolectic
Melitta leporina	x	x	(NT)	LC	Oligolectic
Melitta nigricans	x		(EN)	NA	Oligolectic
Melitta tricincta	x			VU	Oligolectic

**Megachilidae**					
Anthidiellum strigatum	x		(CR)	NA	Polylectic
Anthidium manicatum	x	x	(LC)	LC	Polylectic
Chelostoma campanularum	x	x	(LC)	LC	Oligolectic
Chelostoma florisomne	x	x	(LC)	LC	Oligolectic
Chelostoma rapunculi	x	x	(VU)	LC	Oligolectic
Coelioxys conica	x	x	(LC)	LC	[Parasite]
Coelioxys conoidea	x		(EN)	EN	[Parasite]
Coelioxys elongata	x	x	(LC)	LC	[Parasite]
Coelioxys inermis	x	x	(LC)	LC	[Parasite]
Coelioxys mandibularis	x	x	(NT)	LC	[Parasite]
Coelioxys rufescens	x		(NT)	NT	[Parasite]
Heriades truncorum		x	(CR)	NT	Oligolectic
Hoplitis adunca	x		(EN)	NA	Oligolectic
Hoplitis anthocopoides	x	x	(VU)	VU	Oligolectic
Hoplitis claviventris	x	x	(NT)	LC	Polylectic
Hoplitis leucomelana	x		(CR)	NA	Polylectic
Megachile centuncularis	x	x	(LC)	LC	Polylectic
Megachile circumcincta	x	x	(LC)	LC	Polylectic
Megachile lapponica		x		LC	Oligolectic
Megachile leachella	x	x	(LC)	LC	Polylectic
Megachile maritima	x		(CR)	VU	Polylectic
Megachile nigriventris	x		(EN)	NA	Oligolectic
Megachile versicolor		x		LC	Polylectic
Megachile willughbiella	x	x	(NT)	LC	Polylectic
Osmia aurulenta	x	x	(NT)	LC	Polylectic
Osmia bicornis	x	x	(LC)	LC	Polylectic
Osmia brevicornis	x		(CR)	NA	Oligolectic
Osmia caerulescens	x	x	(LC)	LC	Polylectic
Osmia leaiana	x		(LC)	LC	Oligolectic
Osmia maritima	x		(NT)	NT	Polylectic
Osmia niveata	x	x	(EN)	CR	Oligolectic
Osmia pilicornis	x		(VU)	CR	Polylectic
Osmia uncinata	x		(CR)	VU	Polylectic
Osmia xanthomelana	x		(NT)	NA	Oligolectic
Stelis minuta	x		(EN)	NA	[Parasite]
Stelis phaeoptera	x		(CR)	VU	[Parasite]
Stelis punctulatissima	x		(NT)	VU	[Parasite]
Trachusa byssina	x		(CR)	NA	Oligolectic
**Apidae**					
Anthophora bimaculata	x		(CR)	NA	Polylectic
Anthophora furcata	x		(EN)	LC	Oligolectic
Anthophora plagiata	x		(EN)	RE	Polylectic
Anthophora plumipes	x		(EN)	LC	Polylectic
Anthophora quadrimaculata	x	x	(LC)	LC	Polylectic
Apis mellifera	x	x	(LC)	NA	Polylectic
Bombus barbutellus (ST)	x	x	(EN)	EN	[Parasite]

Bombus bohemicus (ST)	x	x	(LC)	LC	[Parasite]
Bombus campestris (ST)	x		(EN)	LC	[Parasite]
Bombus distinguendus (LT)	x		(NT)	CR	Polylectic
Bombus hortorum (LT)	x	x	(LC)	LC	Polylectic
Bombus hypnorum (ST)		x	(CR)	LC	Polylectic
Bombus lapidarius (ST)	x	x	(LC)	LC	Polylectic
Bombus lucorum (ST)	x	x		LC	Polylectic
Bombus muscorum (LT)	x	x	(NT)	LC	Polylectic
Bombus norvegicus (ST)		x		LC	[Parasite]
Bombus pascuorum (LT)	x	x	(LC)	LC	Polylectic
Bombus pratorum (ST)	x	x	(EN)	LC	Polylectic
Bombus ruderarius (LT)	x	x	(EN)	NT	Polylectic
Bombus ruderatus (LT)	x			RE	Polylectic
Bombus rupestris (ST)	x	x	(LC)	LC	[Parasite]
Bombus soroeensis (ST)	x	x	(LC)	LC	Polylectic
Bombus sylvarum (LT)	x		(LC)	EN	Polylectic
Bombus sylvestris (ST)		x	(EN)	LC	[Parasite]
Bombus terrestris (ST)	x	x	(LC)	LC	Polylectic
Bombus vestalis (ST)		x		LC	[Parasite]
Bombus veteranus (LT)	x		(VU)	EN	Polylectic
Epeoloides coecutiens		x		NA	[Parasite]
Epeolus alpinus	x		(NT)	NT	[Parasite]
Epeolus cruciger	x	x	(NT)	LC	[Parasite]
Epeolus variegatus		x	(NT)	LC	[Parasite]
Eucera longicornis	x		(LC)	LC	Oligolectic
Melecta albifrons	x		(CR)	LC	[Parasite]
Nomada fabriciana	x	x	(VU)	LC	[Parasite]
Nomada ferruginata	x	x	(EN)	LC	[Parasite]
Nomada flava		x		LC	[Parasite]
Nomada flavoguttata	x	x	(NT)	LC	[Parasite]
Nomada flavopicta		x	(LC)	LC	[Parasite]
Nomada fucata		x		LC	[Parasite]
Nomada fulvicornis		x	(LC)	LC	[Parasite]
Nomada goodeniana		x	(LC)	LC	[Parasite]
Nomada lathburiana	x	x	(CR)	LC	[Parasite]
Nomada leucophthalma	x	x	(NT)	LC	[Parasite]
Nomada marshamella	x	x	(LC)	LC	[Parasite]
Nomada obtusifrons	x		(VU)	CR	[Parasite]
Nomada panzeri	x	x	(LC)	LC	[Parasite]
Nomada roberjeotiana	x		(EN)	EN	[Parasite]
Nomada ruficornis	x	x	(VU)	LC	[Parasite]
Nomada rufipes	x		(NT)	LC	[Parasite]
Nomada sheppardana		x		LC	[Parasite]
Nomada striata	x		(NT)	NT	[Parasite]
Nomada succincta	x	x		CR	[Parasite]

Table S2: Tables of p-values for models illustrated in Figure 6.

**p-values for A, based on lecty for bees:** Model: OccursIn2000 ~ Feeding				
Df	Deviance	AIC	X^2	p
<none>	179.99	183.99		
1	188.54	190.54	8.54	0.003

**p-values for B, bee species based on DCA2 score:** Model: OccursIn2000 ~ DCA2				
Df	Deviance	AIC	X^2	p
<none>	84.98	90.98		
1	90.69	94.69	5.71	0.017

**p-values for C, bee species based on DCA3 score:** Model: OccursIn2000 ~ DCA3				
Df	Deviance	AIC	X^2	p
<none>	84.98	90.98		
1	90.84	94.84	5.86	0.015

**p-values for D, based on lecty for non-*Bombus*:** Model: OccursIn2000 ~ Feeding				
Df	Deviance	AIC	X^2	p
<none>	163.25	167.25		
1	170.31	172.31	7.06	0.008

**p-values for E, non-*Bombus* bee species based on DCA3 score:** Model: OccursIn2000 ~ DCA3				
Df	Deviance	AIC	X^2	p
<none>	77.92	81.92		
1	81.77	83.77	3.86	0.049

**p-values for F, based on tongue-length of *Bombus*:** Model: OccursIn2000 ~ Tongue				
Df	Deviance	AIC	X^2	p
<none>	11.09	15.09		
1	16.05	18.05	4.96	0.026

**p-values for G, *Bombus* species based on DCA2 score:** Model: OccursIn2000 ~ DCA2				
Df	Deviance	AIC	X^2	p
<none>	6.69	10.64		
1	12.22	14.22	5.58	0.0182

Table S3: Tables of summary output for models illustrated in Figure 6.

	**Estimate**	**SE**	**Z**	**P**
**All bees**				
Intercept	-0.73	0.31	-2.31	0.02
Lecty	1.09	0.38	2.85	0.01
Intercept	0.11	0.25	0.46	0.65
DCA2	-0.18	0.09	-2.06	0.04
Intercept	0.06	0.25	0.24	0.81
DCA3	0.49	0.26	1.85	0.06
**Non-*Bombus***				
Intercept	-0.73	0.31	-2.31	0.02
Lecty	1.01	0.39	2.60	0.01
Intercept	-0.04	0.27	-0.16	0.87
DCA3	0.43	0.25	1.70	0.09
Bombus				
Intercept	0.00	0.71	0.00	1.00
Tongue-length	19.57	4809	0.00	1.00
Intercept	1.32	1.08	1.22	0.22
DCA2	-0.64	0.40	-1.61	0.11

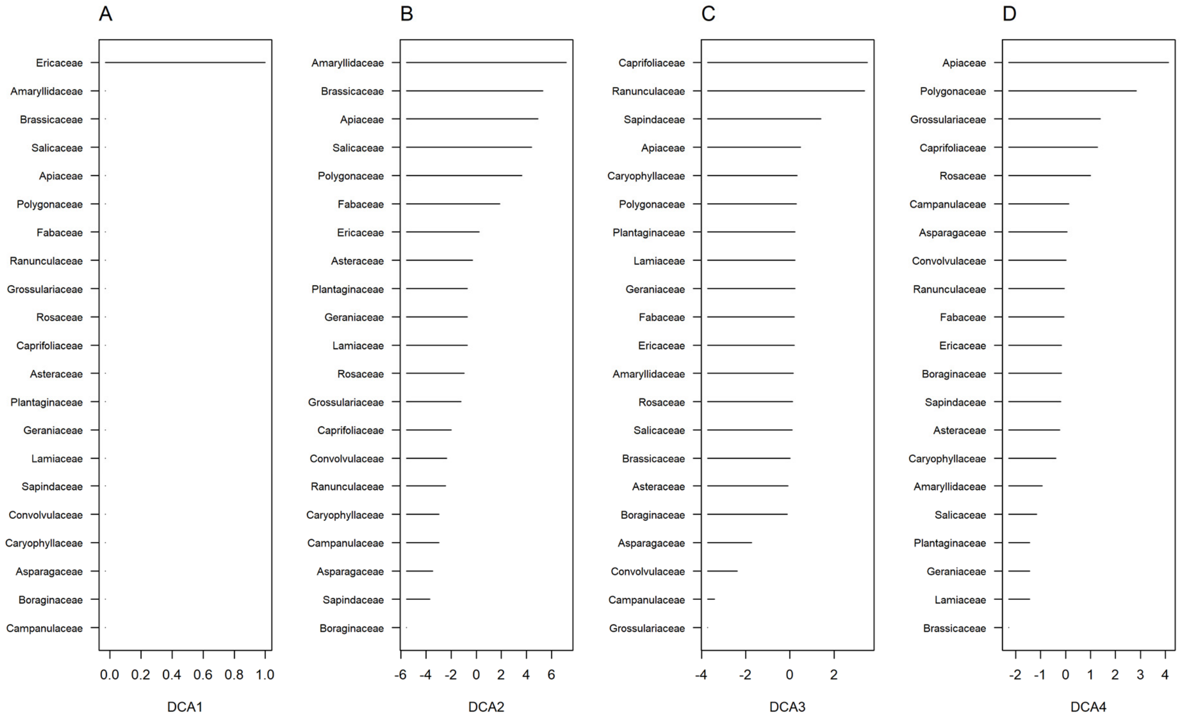

Figure S1: Pollen sampled from females of 80 different bee species collected by Jørgensen or contemporaries in 1900-1919 were divided into four categories based on the composition of the 21 plant families. DCA1 with high occurrence of Ericaceae, etc.

20. Madsen, H.B. Bier. In *Den Danske Rødliste 2019*; Moeslund, J.E., Nygaard, B., Ejrnæs, R., Bell, N., Bruun, L.D., Bygebjerg, R., Carl, H., Damgaard, J., Dylmer, E., Elmeros, M.; et al., Eds.; Aarhus Universitet, DCE—Nationalt Center for Miljø og Energi: Aarhus, Denmark, 2019.
21. Jørgensen, L. Bier. In *Danmarks Fauna*; GEC Gad: København, Denmark, 1921; Volume 25, p. 264.
28. Jørgensen, L. Danske bier. *Flora Og Fauna* **1916**, *22*, 78–90, 129–144.
43. Jørgensen, L. Fortegnelse over de i Danmark Hidtil Fundne Apidae—Strandby Skole. 1921, *unpublished note-book*.
81. Dupont, Y.L.; Damgaard, C.; Simonsen, V. Quantitative historical change in bumblebee (*Bombus* spp.) assemblages of red clover fields. *PLoS ONE* **2011**, *6*, e25172. https://doi.org/10.1371/journal.pone.0025172.
82. Goulson, D.; Hanley, M.E.; Darvill, B.; Ellis, J.S.; Knight, M.E. Causes of rarity in bumblebees. *Biol. Conserv.* **2005**, *122*, 1–8. https://doi.org/10.1016/j.biocon.2004.06.017.
83. Stapel, C. Undersøgelser over humlebier (*Bombus* Latr.), deres udbredelse, trækplanter og betydning for bestøvning af rødkløver (*Trifolium pratense* L.). *Tidsskr. Landbr. Planteavl* **1933**, *39*, 193–294.
